# Immunomodulatory Effects of Bee Venom in Human Synovial Fibroblast Cell Line

**Published:** 2015

**Authors:** Ebrahim Mohammadi, Hossein Vatanpour, Farshad H Shirazi

**Affiliations:** a *Kurdistan Environmental Health Research Center, Kurdistan University of Medical Sciences, Sanandaj, Iran .*; b *Pharmaceutical Sciences Research Center, School of Pharmacy, Shahid Beheshti University of Medical Sciences, Tehran, Iran. *; c *Department of Toxicology and Pharmacology, Shahid Beheshti University of Medical Sciences, Tehran, Iran.*

**Keywords:** Bee venom, Cigarette, Cytokine, Sirt1, Fibroblast-like synoviocytes

## Abstract

As in Iranian traditional medicine, bee venom (BV) is a promising treatment for the rheumatoid arthritis (RA) which is considered as a problematic human chronic inflammatory disease in the present time. Smoking is considered to be a major risk factor in RA onset and severity. The main aim of this study is to investigate the effects of BV on cigarette smoke-induced inflammatory response in fibroblast-like synoviocytes (FLS). Cytotoxicity of cigarette smoke condensate (CSC) and bee venom were determined by the tetrazolium (MTT) method in cultured synovial fibroblastes. The expression of interleukin-1β and sirtuin1 mRNA were analyzed by SYBR green real-time quantitative PCR. Differences between the mean values of treated and untreated groups were assessed by student t-test. Based on MTT assay, CSC and BV did not exert any significant cytotoxic effects up to 40 µg/mL and 10 µg/mL, respectively. Our results showed that interleukin-1β mRNA level was significantly up-regulated by CSC treatments in LPS-stimulated synoviocytes in a dose-dependent manner. Conversely, the expressions of IL-1β and Sirt1 were up-regulated even in lower concentrations of BV and attenuated at higher concentrations. Also, BV attenuated the CSC-induced and LPS-induced inflammatory responses in synovial fibroblasts. Our results support the epidemiological studies indicating pro-inflammatory effects of CSC and anti-inflammatory effects of BV on FLS cell line.

## Introduction

As reported by different epidemiological studies, cigarette smoke is assumed to be the main etiological factor in the pathogenesis of rheumatoid arthritis (1). Cigarette smoke contains numerous compounds such as benzo[a]pyrene, 3-methylcholanthrene, and 2,3,7,8-tetrachlorodibenzo-p-dioxin, as well as free radicals. It has been shown that both cigarette smoke condensate (CSC) and polycyclic aromatic hydrocarbons (PAHs) are able to induce interleukin-1 (IL-1β) and tumor necrosis factor α (TNFα) at mRNA and protein levels in the rheumatoid arthritis cells (2, 3).

Rheumatoid arthritis (RA) is a well-known chronic inflammatory disease associated with hyperplasia of cells in the synovial fluids. RA is characterized by the proliferation of synoviocytes that produce pro-inﬂammatory cytokines which are important in the pathogenesis of RA (4). During synovial hyperplasia of the joint fibroblast-like synoviocytes (FLS) proliferates and forms up to 10-20 layers of cells (5). Fibroblast-like synoviocytes, the key effectors leading to cartilage destruction in the inflamed joint, release a lot of pro-inflammatory cytokines and chemokines (4), among which, IL-1β is the most important cytokine involved in the process of inflammation in RA (6). 

Sirtuin 1 (Sirt1), a mammalian homolog for yeast silent information regulator 2 (SIR2), is an NAD+-dependent deacetylase that belongs to the group III of histone deacetylases (7). It plays a key role in many pathological processes, including aging, stress resistance, apoptosis, inflammation, and autoimmunity (8). The function of Sirt1 is mainly involved in the deacetylation of histones and numerous important transcription factors including nuclear factor-κB (NF-κB) (9). In a previous research, Sirt1 was found to be over expressed in synovial tissues and cells from patients with RA. In addition, Sirt1 is shown to act as a pro-inflammatory protein in synovial cells of RA patients (10).

Bee venom (BV) therapy has been used in traditional medicine for the treatment of arthritis for a long time (11, 12). Anti-arthritis effects of BV have been reported in several arthritis models. Inhibition of nuclear factor kappaB (NF-κB), a critical transcriptional factor responsible for the regulation of inflammation transcription, attributes to melittin as a major constituent of BV (13). Decrease in the pro-inflammatory cytokines (TNF-α, IL-6 and IL-1β) production, cyclooxygenase and phospholipase enzymes, and reactive oxygen species (ROS) were reported to be associated with the anti-arthritis effects of BV (14). Induction of apoptosis in human rheumatoid synovial fibroblasts has also been reported (15).

## Experimental

*Reagents*

DMEM, penicillin, streptomycin, trypsin/EDTA and other cell culture reagents were obtained from Gibco BRL. Lipopolysaccharide (LPS) from Escherichia coli serotype 026:B6 was obtained from Sigma (St. Louis, MO, USA). CSC was purchased from Murty Pharmaceuticals (Lexington, KY, USA), made from Kentucky standard cigarettes (1R3F; University of Kentucky, KY, USA).

*Cell culture*

 Human FLS were cultured in DMEM supplemented with 10% heat-inactivated fetal bovine serum and 50 units/mL penicillin/streptomycin in a humidiﬁed atmosphere of 5% CO_2_ at 37 °C. FLS were detached using trypsin/EDTA and transferred to 96 and/or 6-well plates for experiments. To inhibit any mycoplasma contamination, cells media were continually checked by conditional PCR according to Young *et al.* (16). 

*Cytotoxicity assay*

The cytotoxicity of whole BV and CSC were determined by the ability of living cells to reduce the tetrazolium salt 3-(4,5-dimethylthiazol-2-yl)-2,5-diphenyltetrazolium bromide (MTT) to formazan by the mitochondrial dehydrogenase enzymes. In brief, after culturing in 96-well plates, cells were treated with different concentrations of CSC and BV. Control cells were exposed to 0.1% DMSO and/or sterile normal saline as solvents. The absorbance of each well was determined at wavelength of 570 nm for the dye and 630 nm for the background using a plate reader. The data were then expressed as a relative absorbance at 570 nm.

*RNA Isolation and reverse transcription*

FLSs at passage numbers 3 to 5, were seeded at a concentration of 2 × 10^5^ cells per well in 6-well culture plates. Confluent monolayer cultures in a final volume of 1.5 mL, were then stimulated with LPS (1 μg/mL) for 1 h and treated with 0.4, 4, and 40 µg/mL CSC or 0.1, 1, and 10 µg/mL BV for 24 hours. In another experiments, cells were co-exposed with 40 µg/mL CSC and 10 µg/mL BV simultaneously. Total RNA isolation was performed according to the Chomczynski method using TriPure RNA isolation Solution (Roche, Germany). Extracted RNAs were dissolved in RNase/DNase free water and quantified at 260 nm using a NanoDrop 2000c spectrophotometer (Thermo Fischer Scientific, USA). Besides a check for RNA purity (calculated from the 260 nm/280 nm ratio of higher than 1.6), small and equal aliquots of RNA were used to check the quality of extracted RNA using agarose gel electrophoresis. Genomic DNA was decontaminated in RNA samples by RNase-free DNase I enzyme (Fermentas EN0521). cDNA synthesis was performed exactly as described by the supplier of the maxima first strand cDNA synthesis kit (Thermo Scientific) using 1 µg of RNA per reaction. 

*Quantitative real-time PCR*

After primer set-up, five serials of cDNA were prepared using PCR quality water as diluents to find amplification efficiency of the qPCR in triplicates. Two-step SYBR green RT-PCR amplification was conducted on a StepOne^TM^ real time PCR system (ABI), using QuantiFast SYBR Green PCR Kit (Qiagen). qPCR was performed using the following conditions: initial denaturation for 5 min at 95 °C, denaturation for 30 seconds, annealing /extension for 15 seconds at 60 °C. To verify the specificity of the amplicons, each experiment included a melting curve plus no-template controls. For all comparative gene expressions reactions were performed as triplicates with at least two sets of reproduction. For relative quantification, glyceraldehyde-3-phosphate dehydrogenase (GAPDH) was used as an endogenous control. The lengths of amplified fragments were: GAPDH, 87bp; IL-1β 91bp; Sirt1 86bp (Table 1). To confirm the accuracy of the PCR, products were checked using 1.5% agarose gel electrophoresis visualization. Relative quantitative evaluation of target mRNAs was performed by comparative ΔΔC_T_ method. Reported values have a 95% confidence interval.

**Table 1:** Primer pairs used for PCR of cDNA from FLS cells.

**Product Size**	**Sequence**	**Primer**	**Gene**
**91**	5’-TCC CCA GCC CTT TTG TTG A-3’	IL-1β-For	Interleukin 1β
	5’-TTA GAA CCA AAT GTG GCC GTG-3’	IL-1β-Rev	
**86**	5’-TTC ACC TCG CCG ATC TGC TTC-3’	Sirt1-For	Sirtuin 1
	5’-TCG CAA CTA TAC CCA GAA CAT AGA CA-3’	Sirt1-Rev	
**87**	5’-TGC ACC ACC AAC TGC TTA GC-3’	GAPDH-For	Glyceraldehyde-3-phosphate dehydrogenase
	5’-GGC ATG GAC TGT GGT CAT GAG-3’	GAPDH-Rev	

*Statistical analysis*

Data were expressed as mean ± SEM when distributed normally (cell viability, relative gene expression). Statistical analysis was performed with GraphPad Prism (GraphPad Software, Inc., San Diego, USA; version 5.00). Statistical analysis of real-time PCR result was made using qGENE which is an Excel-based software tool for relative gene expression. The p-values of <0.05 differences were considered significant.

## Results

*Effects of CSC and BV on cell viability*

**Figure 1 (A and B):**
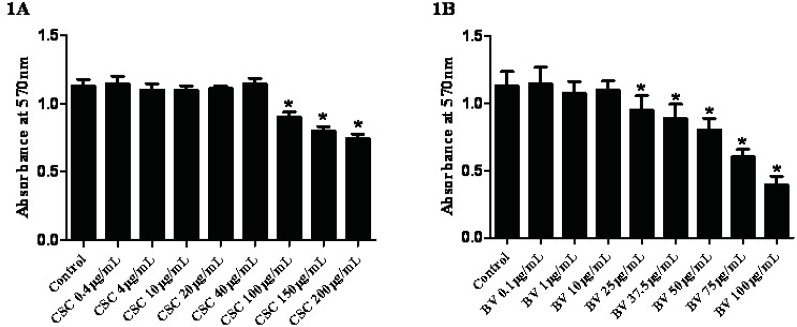
Dose-dependent effects of CSC and BV on synovial cells viability. Viability was estimated by MTT cytotoxicity test. Results are presented as mean +/- standard error. *Represents p-value <0.05 compared to the control.

To determine the effects of CSC on cell viability, FLS cells were exposed to increasing concentrations of CSC for 24 hours and examined using the MTT cytotoxicity test. Neither CSC nor BV exerted any significant cytotoxic effect up to 40 µg/mL for CSC and 10 µg/mL for BV, respectively (Figures 1A and 1B). According to the cell viability and light microscopy observations, sub-toxic concentrations of CSC (≤40 µg/mL) and BV (≤10 µg/mL) were used to analyze the gene expressions.

*Effects of CSC on gene expression*

**Figure 2 (2A and 2B):**
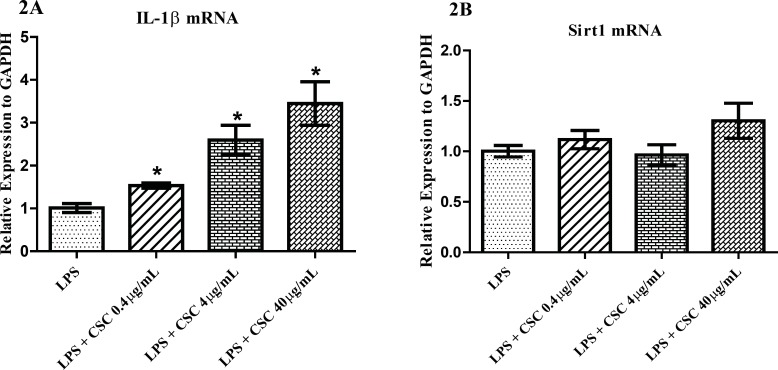
qPCR analysis of the mRNA levels of *IL-1β* and *Sirt1 *after normalization to GAPDH gene. (A) Dose-dependent increase of *IL-1β* gene expression in FLS by CSC. (B) No significant changes of Sirt1 mRNA in FLS. * represents p-value <0.05 compared to the control.

To find the response of synovial fibroblasts to CSC, we performed quantitative PCR for *IL-1β* and *Sirt1* molecules. FLS cells were pre-incubated with 1 µg/mL LPS and then treated with 0.4, 4, and 40 µg/mL CSC for 24 hours. mRNA levels were relatively quantified. As shown in Figure 2A, the expression of *IL-1β* was significantly evaluated to 1.53, 2.59 and 3.44 times that of untreated controls with the increasing concentrations of CSC. The IL-1β mRNA expressions induced by CSC increased in a dose-dependent manner in LPS-stimulated cells (Figure 2A). No change was seen for Sirt1 mRNA after treatment with any concentrations of CSC (Figure 2B).

*Effect of BV on LPS-induced gene expression*

**Figure 3 (3A and 3B):**
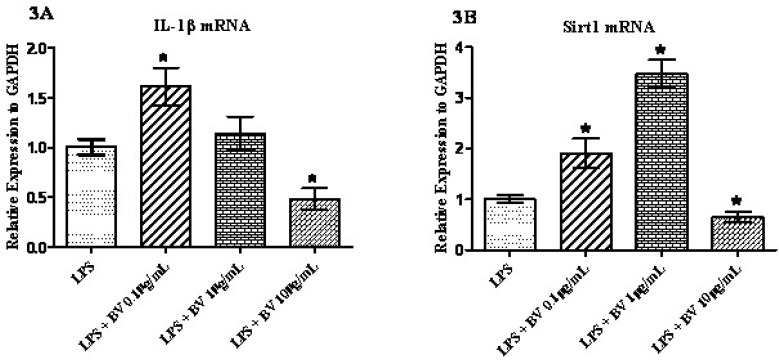
Confluent monolayer cultures of FLS were pretreated with 1 µg/mL LPS for 1 h and then stimulated with three concentrations of BV for 24 h. RNA were quantified by qPCR and normalized to GAPDH. * represents p-value <0.05 compared to the control.

To analyze the influence of BV on synovial fibroblasts, FLS cells were pre-incubated with 1 µg/mL LPS and then treated with three different concentrations of BV for 24 hours prior to the qPCR. As shown in Figure 3A, the expression of *IL-1β* was significantly up-regulated after the exposure of cells to 0.1 µg/mL BV and down-regulated when 10 µg/mL of BV was added to the cell culture media. The amount of expression increased by 1.61 fold in the lower concentrations of BV and decreased by 0.48 fold at the higher concentrations of BV. Statistically no significant difference was seen for the concentration of 1 µg/mL BV (Figure 3A). Interestingly, the expression of *Sirt1* in exposed cells increased following the concentrations of 0.1 µg/mL and 1 µg/mL BV and decreased following treatment with 10 µg/mL BV (Figure 3B). As depicted in the Figure 3B, a 3.47 fold increase in *Sirt1* mRNA was also noted after the exposure of cells to the 1 µg/mL of BV.

*Modulation of CSC gene response by BV*

**Figure 4 (4A and 4B):**
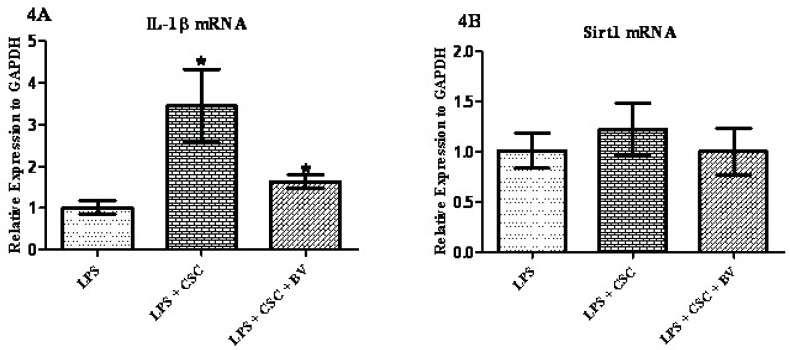
Confluent monolayer cultures of FLS were pretreated with 1 µg/mL LPS for 1 h and then stimulated with three concentrations of BV for 24 h. Gene expressions were quantified by qPCR and normalized based on GAPDH. * represents p-value <0.05 compared to the control.

To examine whether BV can modulate the immunotoxic effects of CSC in FLS cells 1 µg/mL LPS was added to the cell media for 24 hours and then treated with 40 µg/mL CSC alone or in combination with 10 µg/mL BV for 24 hours. As shown in Figure 4A, BV has significantly attenuated the CSC-induced *IL-1β* mRNA by 50%, although *IL-1β* mRNA was still higher in BV exposed cells than LPS-treated cells. Different results were obtained for *Sirt1* gene expression; no significant changes were made by CSC alone or in combination with BV in the levels of *Sirt1* mRNA. 

## Discussion

One of the most important components of the intimal lining layer of the healthy synovium, is FLS, which cellular characteristics can changed under different environmental conditions. One of the important pathologic features of FLS is its ability to secrete cytokines like IL-1. Indeed, Sirt1 protein and gene expression is increased in synovial tissues and cells of RA patients suggesting an important role for Sirt1 in the pathogenesis of RA (10).

The idea behind the current research is to find a link between the most important risk factor of rheumatoid arthritis disease and the inflammatory response in normal FLS using gene expression analysis. To achieve this aim, expression of inflammatory cytokines *IL-1β* and *Sirtuin 1* genes were assessed by quantitative real-time PCR. The gene expression of FLS obtained from RA patients significantly distorts in the higher passages (17). That is why we have investigated the gene expression patterns during the passage 3 and passage 5. Mycoplasma is a microorganism that sometime contaminates cell banks and affects the expression of some genes and proteins (16). Surprisingly, this microorganism has been defined as a cause of RA development before (17). That is why we had to routinely check the FLS cells using the most reliable PCR test applied for this special kind of contamination. Based on the viability assay, we have found that doses of up to 40 µg/mL were the most optimal doses to treat the cells with CSC (FIgure 1A). This is consistent with other *in-vitro* assays on the determination of the sub-toxic doses of CSC in different cell lines (19). It has been reported that concentrations greater than 10 µg/mL of BV may lead to the disintegration of FLS in culture (20). Based on these data and our MTT assay (Figure 1B), it seems that the sub-toxic concentrations of BV should be within the range of up to 10 µg/mL. This is also similar to the previous experiments made in synovial fibroblasts of patients with rheumatoid arthritis (15). Therefore the gene expression analyses were designed at three concentrations of BV (Figures 3 and 4). 

The first description of the association between cigarette smoke and RA dates back to 1987 (21). Cigarette smoke is a complex mixture containing more than 4000 chemicals including some inflammatory agents. It has also been shown that CSC up-regulates gene expression of some inflammatory cytokines including *IL-1β *(3). 3-methylcholanthrene, benzo[a]pyrene, and 2,3,7,8-tetrachlorodibenzo-p-dioxin are some of the ingredients of cigarette smoke. The expression of *IL-1β* has been shown to be increased by these chemicals in RA patient derived SV40 T antigen-transformed human fibroblast-like synoviocyte line MH7A (2). In the present study a dose-dependent increase was seen for *IL-1β *mRNA. LPS-induced production of prostaglandin E2 and nitric oxide decreased it in Raw 264.7 cells at concentrations of 0.5–5 μg/mL BV (13). In contrast, a significant increase in the mRNA level of several pro-inflammatory genes was seen with no changes in NF-κB activation when FLS were exposed to 0.5 or 5 μg/mL of BV (20). As shown in Figures 3 and 4, up-regulation and down-regulation of *IL-1β *mRNA are observed for the lower concentration (0.1 µg/mL) and also higher concentration of BV (10 µg/mL), respectively. Surprisingly, Hamedani and co-workers reported a stimulatory effect of BV at concentrations of 0.05 μg/mL and an inhibitory effect of BV at concentrations lower than 0.05 μg/mL when the human monocyte cell line (K562) was treated with BV (22).

Up to now, two sets of completely different results have been reported on the role of Sirt1 in inflammation; some researchers have reported an anti-inflammatory role of this protein and others reported a completely different role for Sirt1. The conclusions of the former were based on the activation of Sirt1 by resveratrol and reduction of inflammatory response in fibroblasts or human rheumatoid cell line, MH7A (23, 24). The latter obtained the same response by using different sirtuin inhibitors (25, 26). In addition, constitute overexpression of *Sirt1* has been shown to have anti-apoptotic and pro-inflammatory effects in FLS (10), while *Sirt1* over-expression suppress the NF-B and inflammation in osteoclasts (27). This inconsistency has never been seen before in other chronic inflammatory conditions like COPD where only the anti-inflammatory function of Sirt1 has been reported (28, 29). Although the exact role of Sirt1 in inflammation is not yet clear, the results of present study support the pro-inflammatory effects for this histone deacetylase. As seen in Figure 2B, no significant changes were detected when FLS was exposed to different concentrations of CSC. These data does not support a role for CSC on transcriptional level of sirtuin. The result was different when MonoMac6 cells from COPD patients were used, in which CSC decreased Sirt1 level in a dose dependent manner (30). Our data showed that with an exception, the trend for *Sirt1* mRNA is the same as *IL-1β *mRNA; the lower concentrations of BV (0.1 µg/mL) increases the transcriptional levels of *Sirt1*, but higher concentrations of BV (from 10 µg/mL) deceases *Sirt1* mRNA (Figure 3B). As is shown in Figure 3B, the concentration of 1 µg/mL BV augmented the expression of *Sirt1*, which can be interpreted as the immunomodulatory effects of BV on immune response in FLS and this report may confirm the therapeutic effects of BV on RA and other immune-related disorders (31). 
